# Long‐term ecological research and the COVID‐19 anthropause: A window to understanding social–ecological disturbance

**DOI:** 10.1002/ecs2.4019

**Published:** 2022-04-08

**Authors:** Evelyn E. Gaiser, John S. Kominoski, Diane M. McKnight, Christie A. Bahlai, Chingwen Cheng, Sydne Record, Wilfred M. Wollheim, Kyle R. Christianson, Martha R. Downs, Peter A. Hawman, Sally J. Holbrook, Abhishek Kumar, Deepak R. Mishra, Noah P. Molotch, Richard B. Primack, Andrew Rassweiler, Russell J. Schmitt, Lori A. Sutter

**Affiliations:** ^1^ Institute of Environment and Department of Biological Sciences Florida International University Miami Florida USA; ^2^ Institute of Arctic and Alpine Research and Environmental Studies Program University of Colorado Boulder Colorado USA; ^3^ Department of Biological Sciences Kent State University Kent Ohio USA; ^4^ The Design School Arizona State University Tempe Arizona USA; ^5^ Department of Biology Bryn Mawr College Bryn Mawr Pennsylvania USA; ^6^ Department of Natural Resources and the Environment University of New Hampshire Durham New Hampshire USA; ^7^ Institute of Arctic and Alpine Research University of Colorado Boulder Colorado USA; ^8^ National Center for Ecological Analysis and Synthesis University of California Santa Barbara Santa Barbara California USA; ^9^ Department of Geography University of Georgia Athens Georgia USA; ^10^ Department of Ecology, Evolution and Marine Biology University of California Santa Barbara Santa Barbara California USA; ^11^ Department of Environmental Conservation University of Massachusetts Amherst Amherst Massachusetts USA; ^12^ Department of Biology Boston University Boston Massachusetts USA; ^13^ Department of Biological Science Florida State University Tallahassee Florida USA; ^14^ Warnell School of Forestry and Natural Resources University of Georgia Athens Georgia USA

**Keywords:** ecosystems, feedback, LTER, press, pulse, recovery, reorganization, resilience

## Abstract

The period of disrupted human activity caused by the COVID‐19 pandemic, coined the “anthropause,” altered the nature of interactions between humans and ecosystems. It is uncertain how the anthropause has changed ecosystem states, functions, and feedback to human systems through shifts in ecosystem services. Here, we used an existing disturbance framework to propose new investigation pathways for coordinated studies of distributed, long‐term social‐ecological research to capture effects of the anthropause. Although it is still too early to comprehensively evaluate effects due to pandemic‐related delays in data availability and ecological response lags, we detail three case studies that show how long‐term data can be used to document and interpret changes in air and water quality and wildlife populations and behavior coinciding with the anthropause. These early findings may guide interpretations of effects of the anthropause as it interacts with other ongoing environmental changes in the future, particularly highlighting the importance of long‐term data in separating disturbance impacts from natural variation and long‐term trends. Effects of this global disturbance have local to global effects on ecosystems with feedback to social systems that may be detectable at spatial scales captured by nationally to globally distributed research networks.

## INTRODUCTION

Ecosystems worldwide are influenced by human activities at local‐to‐global scales due to modifications of land, water, and atmosphere. Direct effects of anthropogenic activities, such as increasing nitrogen (N) deposition, species relocations, natural resource depletion, and climate change, can cause cascading indirect effects through ecosystems (e.g., changes in productivity, habitat and water quality, wildlife movements, and food webs). The health and well‐being of human populations are also affected directly by disturbances (e.g., hurricanes and wildfires) and indirectly when ecosystem services are disrupted (e.g., water availability and access to parks). Although human‐driven disturbance is a common regulator of ecosystem dynamics at all spatiotemporal scales, the complex feedback among people and ecosystems can complicate efforts to manage social–ecological systems for resilience (Gaiser et al., 2020).

The abrupt change in human activity associated with the COVID‐19 pandemic represents a distinct, pulsed shift in human‐driven disturbance that interrupted some of the continual pressed effects of humans on the land, water, and atmosphere. The term “anthropause” was coined to refer to a period of “considerable slowing of modern human activities,” specifically those observed or anticipated as a result of COVID‐19 mitigation (Rutz et al., 2020). The unprecedented confinement of nearly two‐thirds of the global population provides a “global human confinement experiment” (Bates et al., 2020; Corlett et al., 2020) that allows us to study the beneficial and harmful effects of human presence and mobility on urban, terrestrial, freshwater, and coastal/marine ecosystems. Globally distributed environmental measurements and sensors are likely to capture multiple effects of the anthropause to varying degrees, with effects ranging from undetectable or subtle to significant disruptions in ecosystem dynamics. This range of potential impacts of the anthropause could provide valuable insight into the complex feedback among ecosystems, societies, and disturbance drivers (Stokstad, 2020). Additionally, whether the anthropause leads to long‐lasting changes in the way that humans interact with ecosystems has ramifications for resilience planning. For instance, in a study of human behavior in Israel, postpandemic respondents showed heightened concern about climate change, recycling, and consumption compared to the prepandemic population (Tchetchik et al., 2021). Whether behavioral consequences of these concerns will persist has yet to be explored. Explorations of data from distributed ecological observatories that conduct long‐term ecological research may be useful for addressing key questions about disturbance in social–ecological systems through the global anthropause experiment of the COVID‐19 pandemic.

**FIGURE 1:**
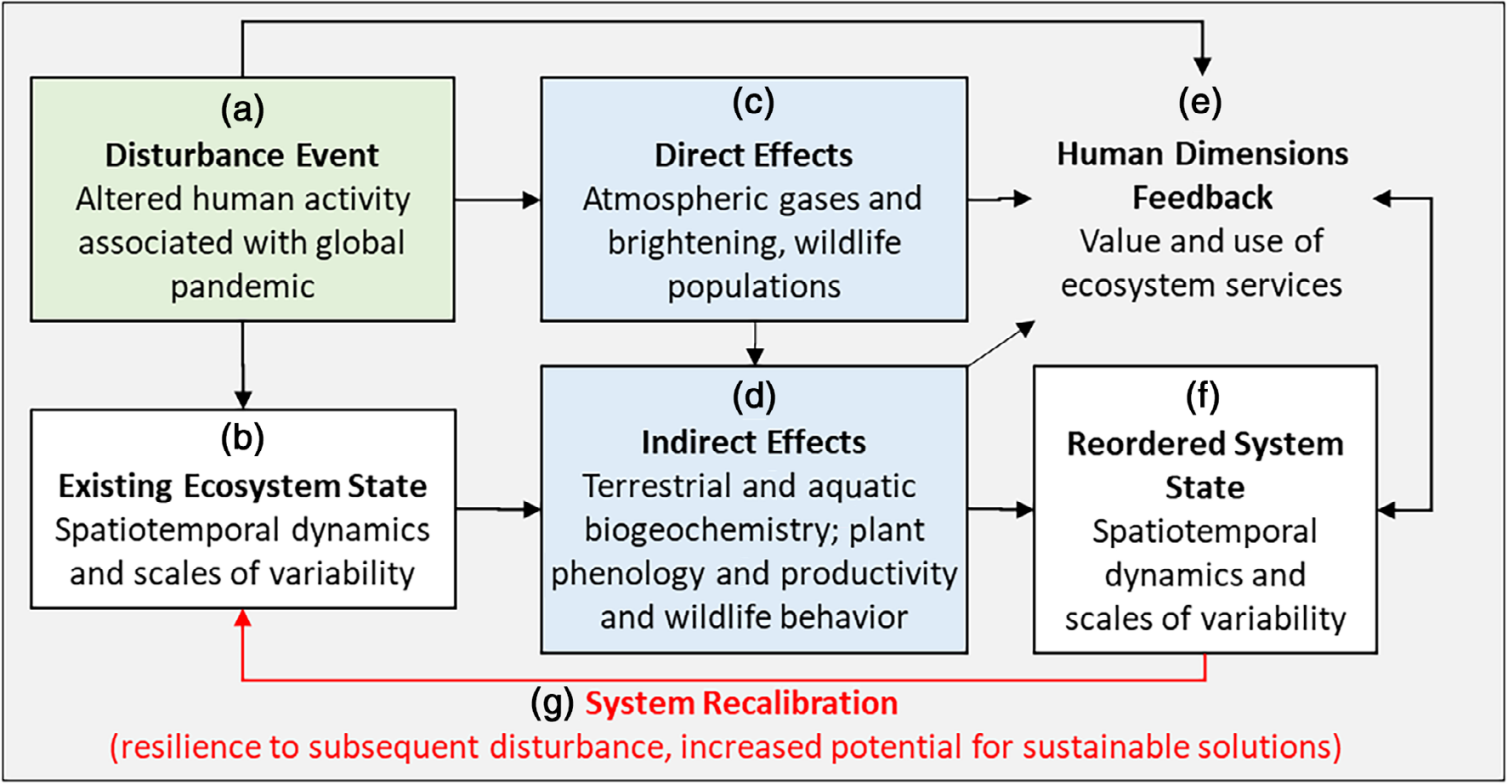
A framework for understanding the pause in human activity resulting from a global pandemic as a disturbance event (a) that disrupts the existing ecosystem state dynamics (b) through direct and indirect effects on air, water soils, and biota (c, d) and via social–ecological feedback (e) that results in a reordered ecosystem state with different spatiotemporal dynamics (f). A key attribute of long‐term ecological research is the ability to capture whether this reorganization occurs, and if it does, how it affects resilience to subsequent disturbance and the potential for sustainable solutions (g). Simplified from Gaiser et al. (2020)

The U.S. Long Term Ecological Research (LTER) Network is a collection of 28 environmental research sites dedicated to documenting and understanding long‐term changes in ecosystem dynamics using hypothesis‐driven approaches in urban, terrestrial, freshwater, coastal, and marine ecosystems. In particular, comparative analyses of multidecadal LTER data have provided insight into how disturbances regulate ecosystems through both slow, continuous (press) and abrupt, short‐term (pulse) changes, including those driven by coupled social–ecological system interactions (Collins et al., 2011; Likens, 1989; Peters et al., 2011). In this paper, we considered how data from the LTER network and other similar distributed observatories could be used to examine how social–ecological interactions responded to the anthropause. We investigated effects of the anthropause using a social–ecological framework based in disturbance ecology theory (Gaiser et al., 2020; Grimm et al., 2017 ; Figure 1), as well as deep knowledge gained from long‐term ecological research. The framework's components and feedback, explained in detail by Gaiser et al. (2020), were populated with the dynamics expected to occur in response to the disturbance event of the anthropause (Figure 1a), characterized by a pulse of disrupted human activity associated with the global pandemic. Disruptions linked to the socioeconomic disturbance of the COVID‐19 pandemic have included reduced agricultural production, vehicle and industrial emissions, and increased use of natural areas (for fishing, poaching, and recreation) (Diffenbaugh et al., 2020). Quantifying the influence of these short‐term disturbances and potential long‐term changes caused by the anthropause requires understanding long‐term variance in ecosystem structure and processes (Figure 1b). Most ecosystems are on a trajectory of change from other prior and ongoing press disturbances and are subject to oscillations due to weather and climate (Kominoski et al., 2018). Anthropause‐induced changes, therefore, may be characterized by the magnitude and duration of signal relative to noise accounting for trends as indicated in long‐term datasets (Jentsch & White, 2019). These spatiotemporal dynamics are well characterized by measurements across the LTER Network's core areas of disturbance patterns, primary production, population dynamics, movement of organic matter, and mineral cycling (Waide & Kingsland, 2021). We hypothesized that direct and indirect signals of the anthropause might be detected in these LTER time series, including direct effects such as increased atmospheric brightening resulting from reduced emissions at local to global scales, and increased wildlife territories and movements due to reduced automobile traffic (Figure 1c). Indirect effects of the anthropause on ecosystems (Figure 1d) might include changing nutrient deposition and loading resulting from atmospheric and land‐use changes that may cascade to influence primary productivity, and altered food webs due to resultant trophic cascades or changing fishing/harvesting. Human dimensions of disturbance, including direct and indirect effects on social systems and feedback to and from ecological systems, are also addressed at LTER sites with a substantial human footprint (Waide & Kingsland, 2021). Ecosystem services may change as a result of disturbance, and those services may be appreciated, used, or valued differently by people (Figure 1e). These disturbance effects and their interactions with human behavior may result in a reordered ecosystem states with spatiotemporal dynamics that reflect disturbance legacies (Figure 1f). An important attribute of this conceptual framework is the recognition that a reordered ecosystem state, should it occur, may be more or less resilient to the next disturbance, partly depending on societal actions that ameliorate negative disturbance impacts. Resilience, a measure of the ecosystem's capacity to absorb disturbance and recalibrate (Figure 1g) while retaining existing function, structure, and feedback (Walker et al., 2004), is uniquely captured by long‐term social–ecological research (Gaiser et al., 2020).

In this paper, we examined the various pathways through which social–ecological systems might respond to the anthropause. We used our conceptual framework to examine the possible direct and indirect effects (Figure 1c,d) that the anthropause may have on (a) biogeochemical responses resulting from regional reductions or redistribution of human‐derived pollutants in the atmosphere and (b) plant, wildlife, and agricultural responses driven by these biogeochemical changes and altered land‐use activities such as recreational or commercial angling/hunting activities and farming. We then discuss possible feedback of altered human dimensions on ecosystems (Figure 1e), such as resulting from economic stress, inequitable burdens/environmental justice, resource use and policy change, and/or altered perceived or real values of ecosystem services. By exploring the anthropause as a pulse disturbance, we may gain a deeper understanding of the sensitivity of social–ecological systems to changing human activities, the benefits of mitigating human impacts, and the social–ecological consequences of social and economic injustice that this pandemic has exposed.

## EFFECTS OF THE ANTHROPAUSE ON ECOSYSTEMS

**FIGURE 2:**
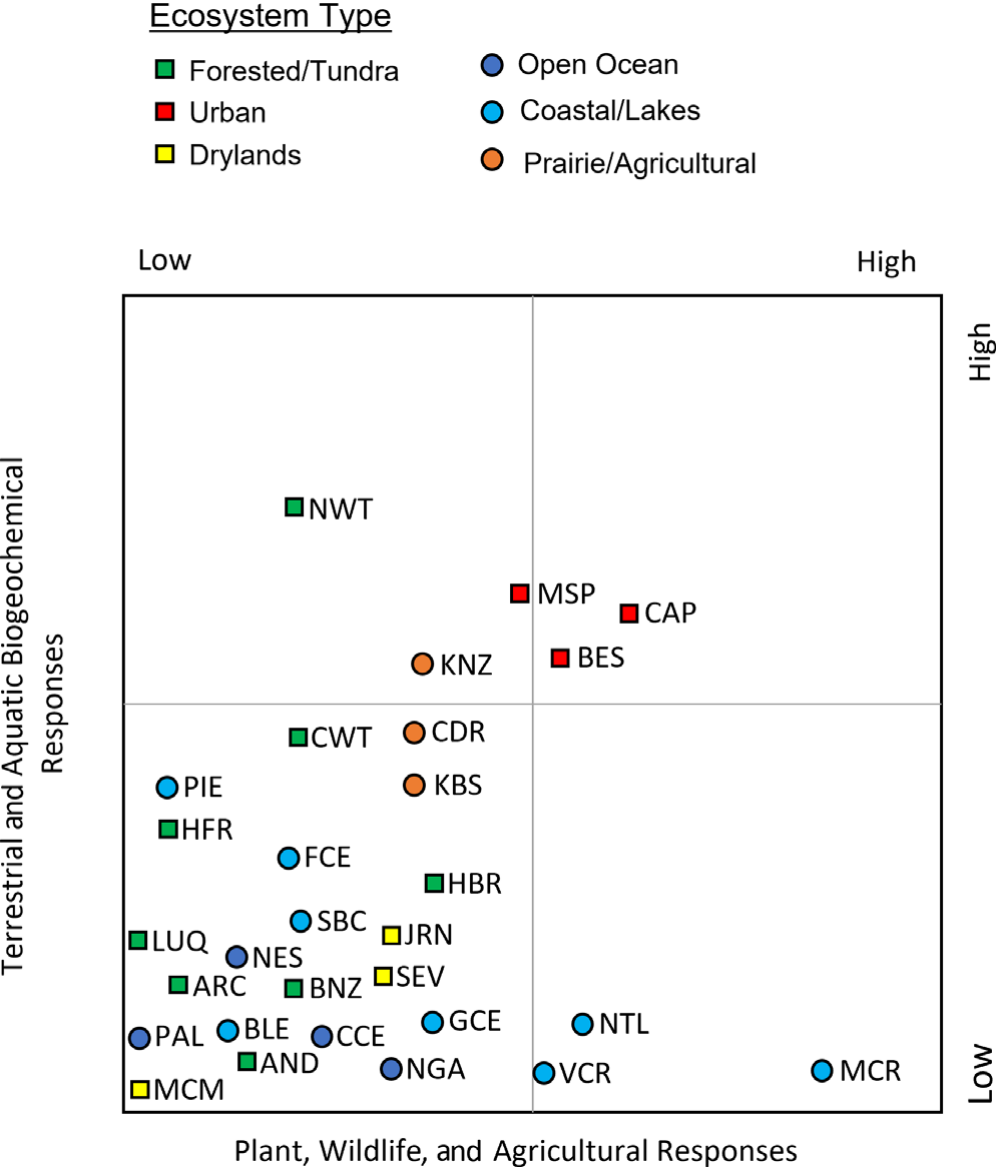
Anticipated effects of the anthropause disturbance on terrestrial and aquatic biogeochemistry (*y*‐axis) and plant, wildlife, and agriculture (*x*‐axis) at U.S. Long Term Ecological Research network sites (see Table 1 for abbreviations and detailed rationale for the qualitative placement of sites)

To illustrate the potential ways that LTER research may be used to characterize effects of the anthropause, we arrayed the LTER sites along axes of terrestrial and aquatic biogeochemical responses, as well as plant, wildlife, and agricultural responses from forested/tundra, urban, desert, open ocean, coastal/lake ecosystems, and prairie/agricultural biomes (Figure 2). The placement of sites along these two axes was based on interviews of the principal investigators of each site who were asked about these expected responses and provide qualitative expectations (described in more detail in Table 1). Because the LTER network includes an array of ecosystem types, we can learn about not only the sensitivities of individual ecosystems to particular attributes of the anthropause but also how the existing ecosystem state, including its disturbance regime, influenced responses to the anthropause. Below, we discuss these expectations for LTER sites, providing insight into how they might inform interpretation of changes in long‐term datasets from other locations in similar ecosystems. Further, we use our conceptual framework (Figure 1) to illustrate and describe three case studies examining the effects of the anthropause.

**TABLE 1:** Expected impacts of the anthropause on the Long Term Ecological Research sites depicted in Figure 2 based on interviews of site principal investigators

Code	Site	Expected impacts
AND	Andrews Forest	Local effects minimal relative to fire but potential effects of increased local recreational traffic on wildlife
ARC	Arctic	Remote, global impact too low and transient; potential local wildlife and air quality effects of reductions in traffic on a major pipeline supply road
BES	Baltimore Ecosystem Study	Less traffic, more outdoor activities, use of parks, subtle impacts on urban ecosystems, substantial impacts on air quality
BLE	Beaufort Lagoon Ecosystem	Remote, global impact too low and transient
BNZ	Bonanza Creek	Remote, global biogeochemical impacts will be low; increased outdoor recreation (ATV and snow machines) and hunting might affect wildlife
CCE	California Current Ecosystem	Pelagic upwelling ecosystem with possible top‐down fishing impacts
CDR	Cedar Creek Ecosystem	Possible impacts on farming, wildlife, air, and water quality
CAP	Central Arizona‐Phoenix	Less traffic, more outdoor activities, use of parks, subtle impacts on urban ecosystems, substantial impacts on air quality
CWT	Coweeta	Biogeochemical responses to atmospheric change that could affect vegetation, wildlife, and stream ecology
FCE	Florida Coastal Everglades	Biogeochemical responses to atmospheric change and water management alterations
GCE	Georgia Coastal Ecosystems	Limited local effects, some impacts to wildlife populations
HFR	Harvard Forest	Subtle effects of air quality
HBR	Hubbard Brook	Effects of increased visitation on wildlife, subtle air quality change
JRN	Jornada Basin	Local effects of increased visitation
KBS	Kellogg Biological Station	No changes in experimental treatments; regional changes in agriculture intensity and site traffic
KNZ	Konza Prairie	Changes in agriculture and fire management related to pandemic
LUQ	Luquillo	Local impacts minimal (reduced traffic and visitation to recreation areas), possible biogeochemical impacts of global atmospheric changes
MCM	McMurdo Dry Valleys	Remote, global impact too low and transient
MCR	Moorea Coral Reef	Top‐down effects of changes in fishing
MSP	Minneapolis‐St. Paul	Less traffic, substantially more outdoor activities, use of parks, subtle impacts on urban ecosystems, substantial impacts on air quality
NWT	Niwot Ridge	Biogeochemical responses to atmospheric change that could affect vegetation, wildlife, and stream ecology
NTL	North Temperate Lakes	Top‐down effects of changes in fishing
NES	Northeast U.S. Shelf	Changes in runoff and air quality impacts
NGA	Northern Gulf of Alaska	Top‐down effects of changes in fishing
PAL	Palmer Antarctica	Remote, global impact too low and transient
PIE	Plum Island Ecosystems	Local effects on biogeochemistry and potential wildlife impacts of increased refuge visitation and traffic
SBC	Santa Barbara Coastal	Effects of changing coastal recreation on wildlife (especially birds)
SEV	Sevilleta	Subtle effects of air quality
VCR	Virginia Coast Reserve	Top‐down effects of changes in fishing and tourism

### Terrestrial and aquatic biogeochemical responses

**FIGURE 3:**
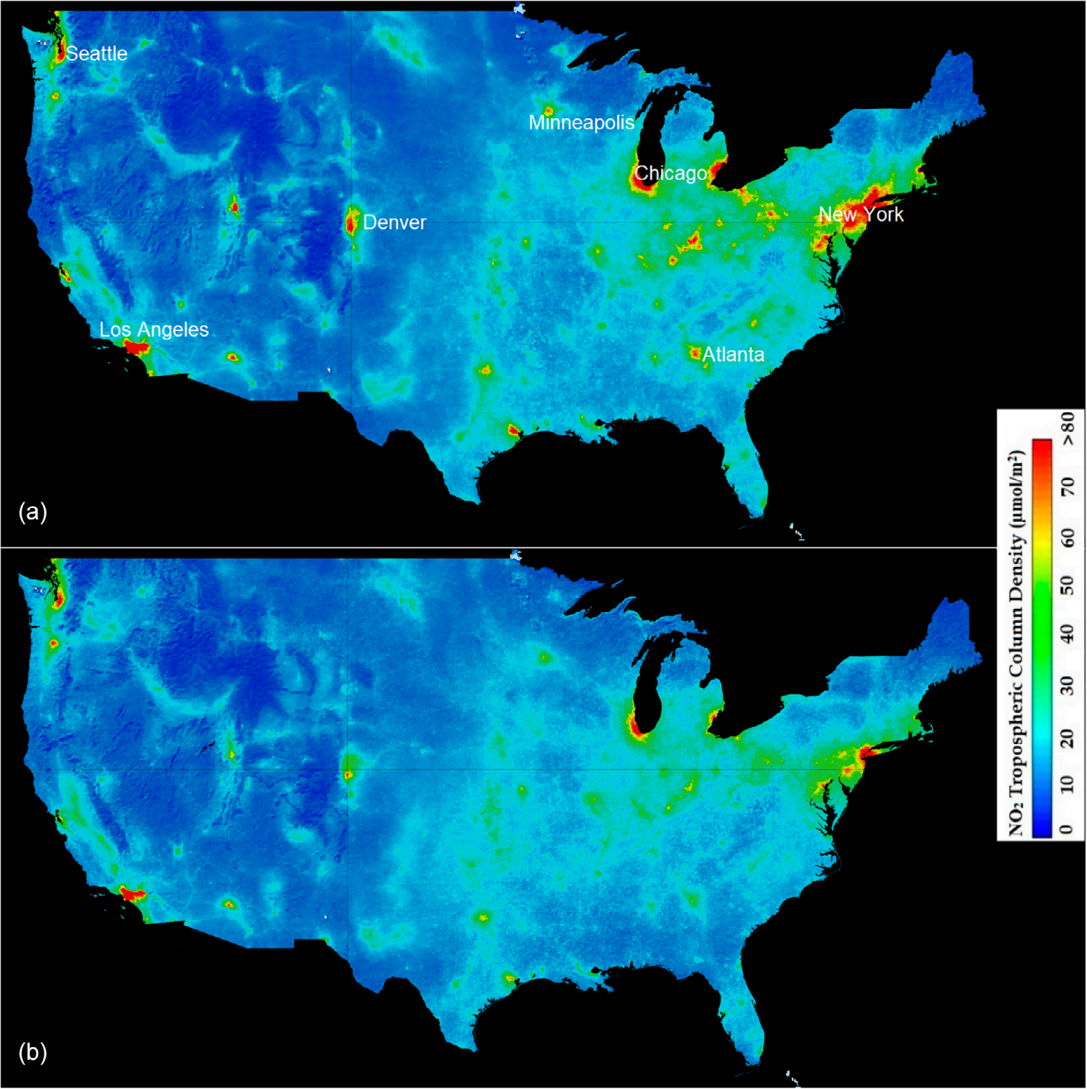
March–April averaged NO_2_ tropospheric column density for the contiguous United States in 2019 (a) and 2020 (b). Source: NO_2_ data were derived from the TROPOspheric Monitoring Instrument onboard European Space Agency's Copernicus Sentinel‐5 precursor satellite. A notable decline in NO_2_ density was observed across major urban centers, presumably due to various lockdown measures. The most noticeable reduction was seen in the northeast United States, the region where COVID‐19 was most prevalent in the early phases of the pandemic.*Note*: Vertical column NO_2_ (daily images) have higher uncertainty over less polluted regions yielding negative values at times. We have averaged NO_2_ concentration over 2 months (March–April) to minimize the variability due to sensor noise

Reduced economic activities and shifts in commuter work patterns due to the COVID‐19 pandemic led to declines in some pollutants, including emissions of NO_x_ and CO_2_ (Ding et al., 2020; Feng et al., 2020; Le Quéré et al., 2020; Mishra et al., 2020; Figure 3), redistribution of others such as fertilizer, and indirect influence on inputs of sunlight and water (Diffenbaugh et al., 2020). Declines or shifts in biogeochemical inputs caused by the anthropause can potentially lead to a range of ecosystem effects that may become evident in LTER datasets in the coming years to decades. Although global CO_2_ emissions declined during the first months of the shutdown in some regions, these may not have sustained impact on global atmospheric CO_2_ concentrations (Zheng et al., 2020). Lowered global emissions, however brief, may interact in urban areas with more sustained localized declines in automobile CO_2_ emissions related to reduce commuter traffic. Potential shifts in pollution also include reduced wastewater discharges in urban work centers accompanied by increases in suburban and exurban areas. Suburban areas are often more reliant on septic waste management and therefore subject to greater nutrient discharge to soils and groundwater. Suburban land use may also have intensified as people remained home and unable to travel, thereby spending more time in yards and gardens to gain food security (Bulgari et al., 2021) and health benefits (Corley et al., 2021). Additionally, the anthropause has been accompanied by an exodus of affluent residents from urban centers to second (or new) homes in suburban and rural areas (Devine‐Wright et al., 2020; Zoğal et al., 2020), which may also shift pollutant sources and resultant biogeochemical hotspots and cycles.

Whether ecosystem effects of the anthropause are in fact realized and functionally significant will depend on the spatial and temporal scales of both the change in inputs and the receiving ecosystems. In effect, the question is whether the disturbance is large enough in magnitude, occurring over a large enough area, and of sufficient duration, given the size of the ecosystem and the rate of the processes occurring therein (I. L. Hale, Wollheim, et al., 2014). For example, with respect to urban trees, the decline in local CO_2_ due to reduced commuter traffic in urban centers may be substantial enough to detect local reductions in urban tree growth (Diem et al., 2006; Fares et al., 2017). By contrast, at broader spatial scales, this CO_2_ disturbance will be quickly dispersed and diluted in the atmosphere, resulting in little change in downwind forests. Gradient studies that incorporate urban core to suburban or exurban areas at urban LTER sites (for instance, Central Arizona‐Phoenix, Minneapolis‐St. Paul, and Baltimore Ecosystem Studies LTER sites in the upper right quadrant of Figure 2) could identify this signal in terrestrial vegetation. Similarly, declines in N deposition may be large near urban areas where impervious surface increase connectivity between atmospheric N deposition and waterways (Lewis & Grimm, 2007) but be quickly diluted further away (Sponseller et al., 2016). Certain N‐sensitive surface waters may respond rapidly and acutely to reduced N deposition with altered primary productivity, microbial respiration, and hydrologic exports (Bettez & Groffman, 2013; R. L. Hale, Turnbull, et al., 2014; McCrackin et al., 2008). Mishra et al. (2020) found that coastal waters off of highly polluted parts of urban India with extremely high greenhouse gas emissions experienced a two‐ to threefold decline in nitrate (NO_3_^−^) during the COVID‐19 lockdown, which decreased premonsoon phytoplankton content in those waters compared to previous years.

While decreased N deposition may reduce nutrient loading, the effect may be countered by elevated fertilizer application to suburban lawns and gardens, or increased use of local septic systems as more people work from home, both of which result in greater amounts of N entering into groundwater and streams during base flow, could begin to modify stream ecosystem processes (Reisinger et al., 2017, 2019). The watersheds of the Plum Island Ecosystems LTER encompass suburban Boston, with many communities still on septic systems (Wollheim et al., 2005), where this signal may become evident in surface water chemistry of headwater streams. However, as water flows further downstream to larger rivers, the watershed area being drained expands, and the effects of urbanization are diluted or attenuated, likely making the signal less detectable (R. L. Hale, Turnbull, et al., 2014).

Realization of ecological effects of biogeochemical changes related to the anthropause will also depend on the timing and magnitude of the disturbance relative to ambient seasonal variation. For example, the onset of shifting work and commuting patterns in 2020 coincided with snowmelt, leaf out, and changing seasons in many parts of North America, when rapid ambient changes in ecosystem processes could obscure perturbations of short duration or smaller magnitude. Multidecadal, continuous time series offer potential to quantify seasonality and detect deviations from expected patterns (Cazelles et al., 2008; Sabo & Post, 2008). Long Term Ecological Research sites with urban or suburban headwater streams may be more likely to show changes over the long term to the COVID pandemic. For example, Saw Mill Brook at the Plum Island Ecosystems LTER drains a predominately suburban catchment with abundant lawns, but no septic systems because homes are on a sewer system that leaves the catchment (Wollheim et al., 2005). This stream has been monitored for 20 years for flow and chemistry. Trends in changing human activity have been difficult to detect due to overriding signal of interannual climate variability (Morse & Wollheim, 2014). But if changes in lawn fertilization rates are sustained beyond the anthropause, altered stream chemistry may become evident in coming years as additional data allow deconvolution of the climate signal.

Here, we explored in greater detail two case studies documenting potential near‐term effects of the COVID‐19 anthropause on biogeochemical cycles. Case Study 1 reveals increased light transmission, brightening, and snowmelt stemming from a decline in particulate emissions at the regional scale—changes that could increase photosynthetically active radiation and gross primary production. Case Study 2 suggests declines in reactive N deposition at regional scales that could affect primary production and N cycling in downwind terrestrial and downstream aquatic ecosystems.

#### Case Study 1: Cascading responses of atmospheric clearing on snowmelt and lake production

Of the many critical ecosystem services that must be maintained during the pandemic, a robust food and water supply is among the most essential. Agricultural production in the semi‐arid western United States relies heavily on mountain snowmelt as a water source (Bales et al., 2006). Efficient management of this water supply relies on relatively accurate forecasts of the timing and magnitude of snowmelt‐driven streamflow. Decades of research have illustrated that solar radiation is the primary driver of mountain snowmelt, providing approximately 75% of snowmelt energy (Bloschl, 1991; Cline, 1997; Skiles et al., 2018). The COVID‐19 pandemic, and associated declines in industrial activity, has caused transient global brightening in which the aerosol loads in the atmosphere have been dramatically reduced (Liu et al., 2021). Cities worldwide experienced some of the highest levels of air quality observed in several decades (e.g., fine and large particulate concentrations in Denver have declined by 40% due to COVID‐19 [Colorado Department of Public Health]). Historically, these pollutants and other aerosols have directly increased the opacity of the atmosphere (i.e., the atmosphere optical depth, AOD) and reduced incident solar radiation on the regional land surface (i.e., dimming). Increases in industrial activity, and associated aerosol loading, in the United States and Europe from the 1950s through 1980s were associated with atmospheric dimming and reductions in incident solar radiation (Wild, 2012). The subsequent decades were associated with background brightening in these regions as emission standards were enhanced (Wild, 2012). These aerosols also have an important indirect cooling effect as they increase the number of water droplets in the atmosphere and therefore increase cloud cover and precipitation (Rosenfeld et al., 2019). In addition, the reduction in atmospheric pollutants associated with the anthropause has resulted in regional decreases in the deposition of light absorbing impurities onto snow and ice, which may have delayed the onset of snowmelt (Bair et al., 2021).

From the perspective of atmospheric clarity, the decline in these pollutants as a result of COVID‐19 has caused dramatic decreases in AOD during the onset of the pandemic and associated lockdowns that represent an acceleration of the decadal trend of overall brightening in most industrialized portions of the world (e.g., Europe and the United States) and a reversal of decadal dimming trends in other parts of the world; for example, in parts of China, AOD has decreased by as much as 10% during the onset of the pandemic (Ding et al., 2020). The implications of a potential 10% increase in downwelling solar radiation to mountain environments in the western United States and globally have not been explored. Complicating efforts to identify pandemic‐related signals in AOD are the impacts of wildfire, which represent a large source of aerosols to the atmosphere that have exhibited mixed trends in recent decades (Doerr & Santin, 2016).

**FIGURE 4:**
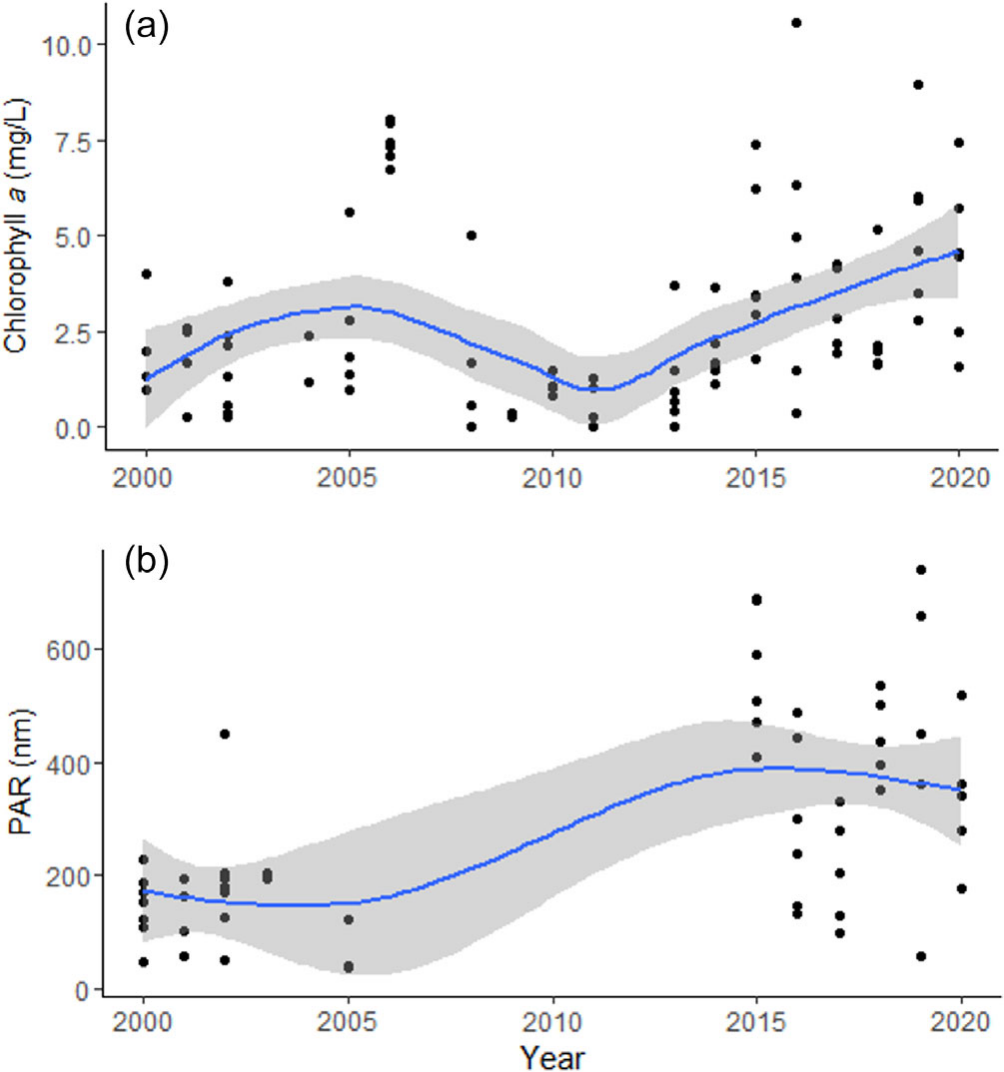
Trends in annual mean (a) chlorophyll *a* (mg/L) and (b) photosynthetically active radiation (PAR; nm) in Green Lake 4 at Niwot Ridge Long Term Ecological Research (LTER). Measurements were taken mid‐lake at a 3‐m depth and up to six sampling events occurred per year although PAR sampling was suspended between 2005 and 2015. A smoothing function has been added to both figures for demonstration. No apparent effect of the anthropause was detectable at annual resolution of the data amid a decadal‐scale trend in both parameters

The transient brightening signal likely reduced snowfall, accelerated snowmelt rates, and shifted peak snowmelt earlier in the year (Appendix S2: Figure S1); pre‐COVID‐19 data and a detailed snowmelt model could be used to isolate the COVID‐19 signal. The AOD, meteorological, and hydrologic measurements would reveal the impacts of COVID‐19 transient global brightening on snowmelt rates. Long‐term research from high‐latitude/altitude locations (e.g., Niwot Ridge, North Temperate Lakes, Bonanza Creek, and Arctic LTER sites) may reveal the extent to which AOD‐associated changes in snowmelt affect phenology, gross primary production, and other ecosystem attributes. To examine this possible response cascade, we examined trends in chlorophyll *a* and photosynthetically active radiation measured since 2000 in Green Lake 4, an oligotrophic lake located at 3500 m above sea level at the Niwot Ridge LTER (Figure 4). Data show increasing chlorophyll *a* since 2013, and highly variable PAR that is higher in the last 5 years than the first 5 years of record. The long‐term trend has been associated with changing ice cover phenology and warming spring temperatures (Christianson et al., 2021; Preston et al., 2016). High interannual variability and an underlying long‐term trend prevent the detection of a COVID‐related effect on the 2020 season. Nonetheless, this case illustrates the importance of long‐term data for interpreting effects of a given disturbance event. Continued data collection may reveal ecosystem changes relevant to prediction of future water availability as clean energy sources and associated with AOD‐induced brightening become more commonplace. Thus, regional long‐term trends superimposed upon interannual variability have important broader impacts in the context of current and future industrial activity. Broader impacts will also occur in the context of providing timely information for water resources management in the western United States.

#### Case Study 2: Response of coastal ecosystems to shifting nutrient loading

**FIGURE 5:**
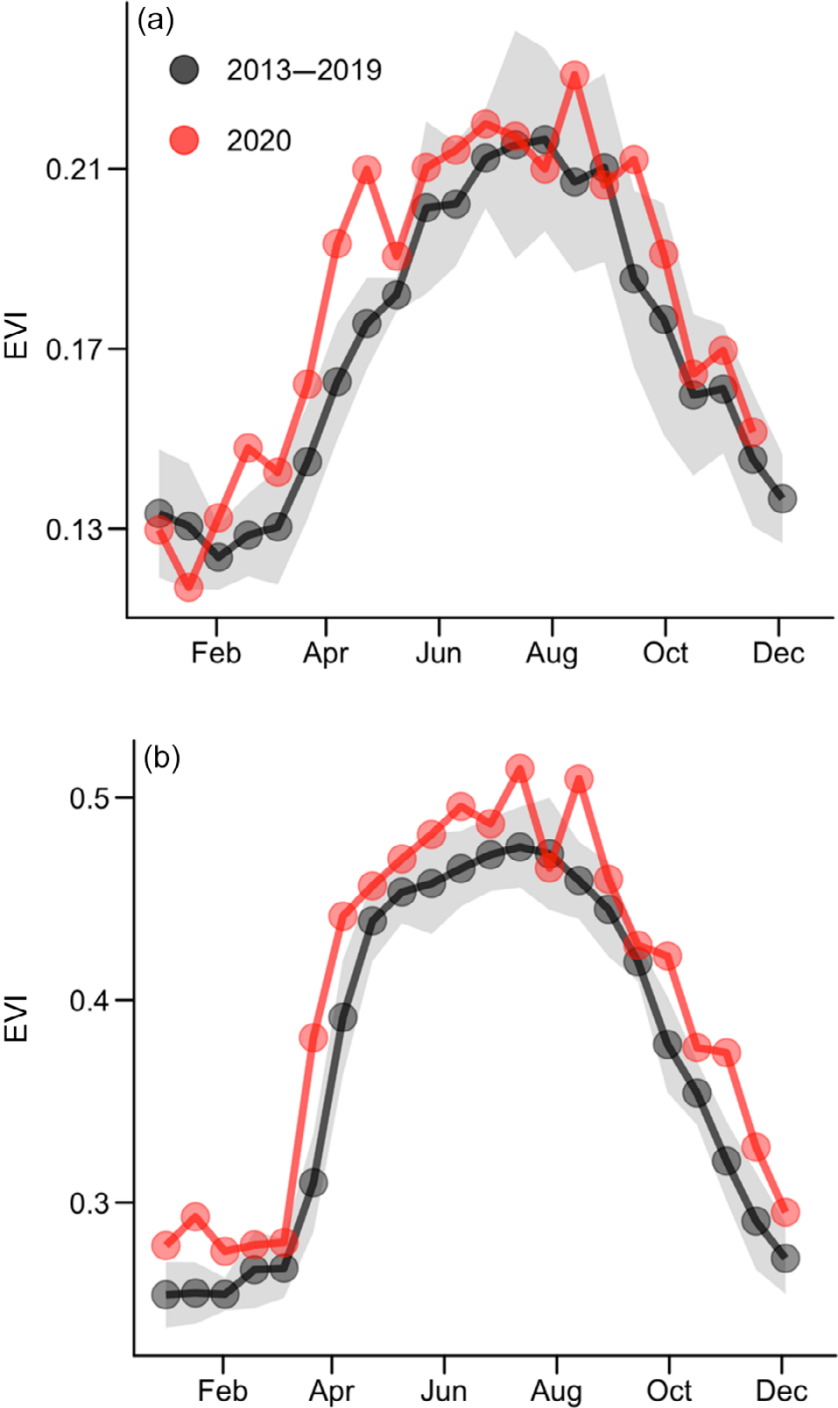
Sixteen‐day mean enhanced vegetation index (EVI) for estuarine and marine wetlands within the Georgia Coastal Ecosystems Long Term Ecological Research (LTER) domain (USFWS National Wetland Inventory) (a). EVI is a well‐recognized index for evaluating vegetation “greenness,” and was derived from NASA MODIS MOD90GA surface reflectance. Wetland MODIS pixels were filtered following O'Connell et al. (2017) to remove intermittent tidal flooding effects on spectral reflectance. (b) Cropland EVI time series sampled from the coastal plain region of Georgia. The uninterrupted green‐up in croplands in spring 2020 is indicative of a reduction in human interventions (e.g., harvesting). Data for 2013–2019 are represented as means (black points) and SDs (gray shaded region)

**FIGURE 6:**
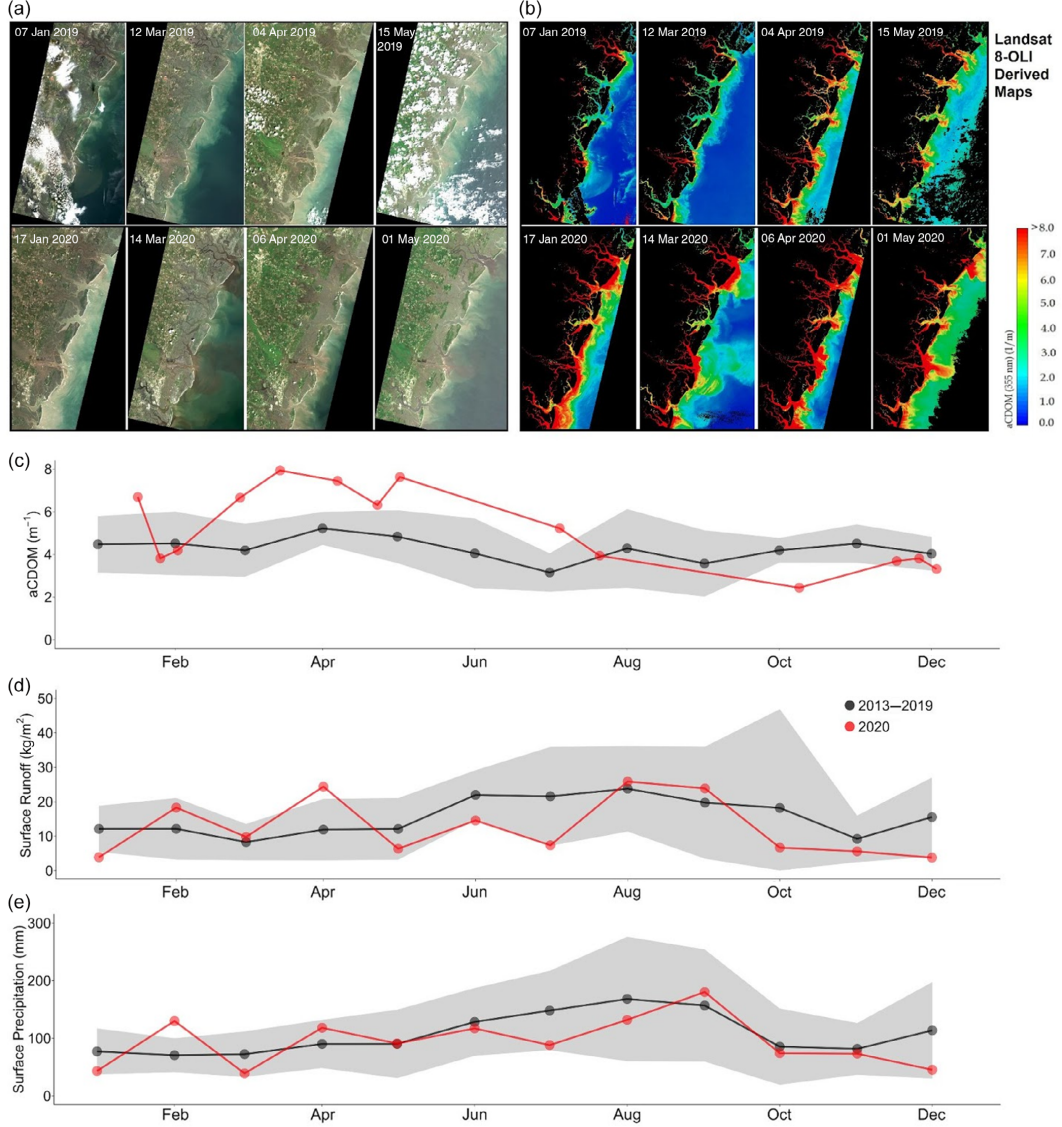
Comparison of (a) monthly true color images and (b) estimates of absorption by colored dissolved organic matter (CDOM [aCDOM]) at 355 nm (m^−1^) in Georgia coastal waters in 2019 and 2020. Time series of (c) Landsat 8 derived a CDOM for the Georgia Coastal Ecosystems Long Term Ecological Research (LTER) domain showing monthly means for the years 2013–2019 compared to 2020. Time series of (d) area‐averaged monthly means (2013–2019) of surface runoff compared to 2020 and (e) monthly means of surface precipitation (2013–2019) compared to 2020 for coastal Georgia. The surface runoff and precipitation data were derived from NASA's MERRA‐2 long‐term global re‐analysis database (MERRA‐2 Model M2TMNXLND v5.12.4). Data for 2013–2019 are represented as means (black points) and SDs (gray shaded region), and 2020 data are represented as red points. Source: Landsat 8‐OLI. The CDOM model (*R* ^2^ = 0.74) used in this study was originally developed for Landsat 5‐TM (Joshi & D'Sa, 2015). However, both Landsat 8‐OLI and Landsat 5‐TM have similar green and red bands (band centers and bandwidths) that were used in the CDOM model, and therefore, we assume the impact of the sensor differences would be minimal in CDOM estimation. Note about uncertainty: The model was developed for Barataria Bay, Louisiana. We have not tuned the model for coastal Georgia because of the lack of in situ data

Conditions in coastal Georgia present a rich field to explore the drivers and states of nutrients and carbon in response to the anthropause. The Georgia Coastal Ecosystems LTER encompasses an area of approximately 1000 km^2^ covering upland (mainland, barrier islands, and marsh hammocks), intertidal (fresh, brackish, and salt marsh), and submerged (river, estuary, and continental shelf) habitats and includes the Altamaha Sound where the Altamaha River debouches (GCE LTER, n.d.). Therefore, the program is well positioned, geographically, to explore anthropause effects on coastal ecosystems that may originate from inland sources. In 2020, “green‐up” of tidal marshes occurred approximately 14 days earlier than the mean of the previous 7 years of observation (Figures 5 and 6). Concentrations of colored dissolved organic matter (CDOM) in the surrounding estuaries were 1.5 times greater than the average of the past 7 years (Figure 6c). Higher precipitation was also observed in early 2020 (Figure 6e), facilitating greater surface runoff (Figure 6d). The higher surface runoff observed in April may have delivered excess nutrients and carbon explaining the increased CDOM and faster rate of green‐up in coastal marshes in 2020 (Appendix S2: Figure S2).

Increased nutrient inputs from increased septic use or agricultural waste (unharvested and decaying crops), or a combination of the two, likely increased nutrients and CDOM in estuaries and rapid green‐up of marsh vegetation. Widespread shelter‐in‐place orders in March–April 2020 interrupted supply chains impacting food security, labor availability, and agriculture system connectivity (Stephens et al., 2020). Farmers were unable to bring their products to markets when many institutional users such as restaurants and schools remained closed. Thus, massive amounts of crops were left unharvested, plowed over, buried, or dumped (Yaffe‐Bellany & Corkery, 2020). Farmland within the coastal plain region of Georgia showed higher greenness during 2020 than previous years (Figure 5b), which could be evidence of unharvested crops. Another source of excess nutrients might be increased use of septic systems, a common source of nutrients to coastal ecosystems (Ngatia et al., 2019; Valiela et al., 2000) as workers shifted to more at‐home use instead of other municipal waste collection systems (North Central Region Water Network, 2021). In a year with record precipitation, such as experienced in Georgia in spring 2020, excess nutrient input septic sources likely resulted in abundant macronutrients and carbon sources to local receiving water bodies.

Although other impacts, such as reduced atmospheric deposition of N (Figure 3), could offset this greater input from agricultural or septic waste, they are not likely to offset the spring green‐up seen in the Georgia Coastal Ecosystems LTER domain because of the relatively low pre‐anthropause atmospheric NO_x_ concentrations measured in this region. The pulse disturbance of increased nutrient loading could increase marsh grass aboveground biomass and/or canopy chlorophyll concentration (Figure 5b) and could have potentially elevated carbon sequestration rates. Experimental studies have shown salt marsh production and associated food webs can rebound from nutrient pulses after 1 year (Deegan et al., 2012; Murphy et al., 2012). Therefore, if the nutrient loading levels return to levels that occurred prior to the anthropause, we would expect the system to return to its previous state.

Field‐collected nutrient data collected during the anthropause to test the above patterns and trends will be available but have not been processed due to laboratory access restrictions related to the COVID‐19 pandemic. Additional long‐term data will be needed to determine whether the extreme greening in 2020 was due to the anthropause, the unusually wet spring, or interactions between these and other drivers. The coastal plain region of Georgia experienced drought conditions at the end of 2019 (Palmer drought severity index of −1.75 during October 2019) preceding the wet spring of 2020 and could have allowed nutrients to build up in the landscape prior to returning precipitation (Huntington et al., 2017). Our hypothesis can be tested by looking at the interaction between nutrient loads and precipitation and runoff rates over the past decade. Long‐term time series from the network of coastal LTER sites (Plum Island Ecosystems, Virginia Coast Reserve, Georgia Coastal Ecosystems, and Florida Coastal Everglades) may indicate whether conditions during the anthropause were anomalous compared with the baseline relationship. Near coastal urban centers, atmospheric N, and other pollutants tend to accumulate over coastlines because of the interaction between low dry deposition rates over water and onshore winds (Loughner et al., 2016). We therefore expect that LTER sites, such as Plum Island Ecosystems LTER in the Boston Metropolitan region, would have experienced a net decrease of atmospheric N associated with the anthropause while also experiencing increased N due to higher N fertilization rates on lawns, and from communities with septic systems (Wollheim et al., 2005). Long‐term data that account for interannual climate variability are essential for distinguishing an anthropause signal. Utilizing a network of long‐term, in situ nitrate sensors for nutrients or eddy covariance flux towers measuring ecosystem CO_2_ exchange between the land surface and the atmosphere may provide insight on long‐term nutrient and productivity effects from the anthropause.

### Wildlife and trophic dynamics responses

Several recent papers summarize potential effects of the anthropause on wildlife (e.g., Bates et al., 2020; Rutz et al., 2020; Zellmer et al., 2020) describing scenarios under which human–wildlife interactions may increase or decrease (e.g., due to greater recreational activities that bring people in contact with wildlife in urban parks as people have more time to get outside given work‐from‐home orders or decreased interactions if entire national parks are closed off from the public). Although all these papers focus on the need for continuous observations during and after the pandemic, Zellmer et al. (2020) also highlight the need for long‐term baseline data in interpreting these observations, and Bates et al. (2020) provide a brief discussion of temporal context. Despite the recognition that long‐term datasets are needed to explore the effects of the anthropause on wildlife, a few studies present evidence based on long‐term data (but see Derryberry et al., 2020). The long‐term nature of population dynamics data collected by all LTER sites provides information on temporal variability that is necessary to confidently attribute changes in wildlife patterns pre‐ and postpandemic to the anthropause, as opposed to other sources of variation (e.g., climate, land‐use change, or natural population fluctuations). Because populations are dynamic systems that are ultimately impacted by a multitude of drivers, an understanding of dynamics in the context of historical drivers and collection of consistent and complementary data become essential in understanding how pulsed, external changes affect these systems (Bahlai & Zipkin, 2020). At LTER sites, the legacy of study of key populations provides a best‐case scenario for providing an understanding of internal dynamics, ongoing relationships of populations with drivers, and concurrent collection of relevant contextual data. Furthermore, consistency among long‐term study sites in the processes and response variables explored facilitates the search for generality, which is a strength of the LTER approach (e.g., Burkepile et al., 2020). Almost all effects on fishes and wildlife would be expected to be mediated through changes in human activity, and we would expect human activities to have different impacts on different systems. In Case Study 3, we combined data from multiple LTER sites to illustrate how long‐term ecological research can help to identify the influences of different drivers of taxa subject to pre‐ and postpandemic fisheries pressure.

#### Case Study 3: Cascading effects of changes in commercial and small‐scale fishing due to the anthropause

**FIGURE 7:**
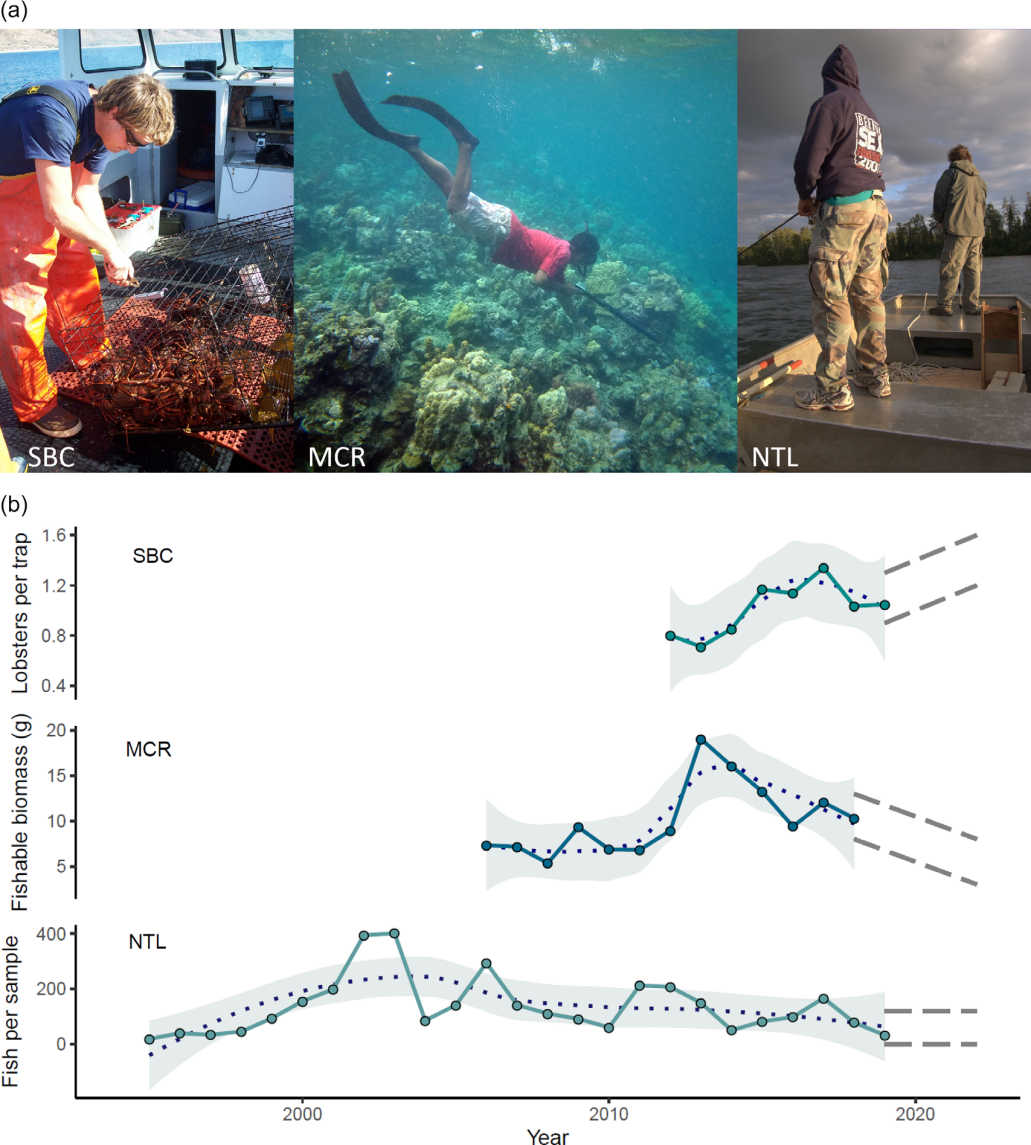
(a) Fishing practices at three different Long Term Ecological Research (LTER) sites including commercial lobster trapping at the Santa Barbara Coastal LTER (SBC, photo by Jono Wilson), local spearfishing from shore at Moorea Coral Reef LTER (MCR, photo by Jean Wencélius), and recreational angling from a boat at North Temperate Lakes LTER (NTL, photo by Noah Lottig). (b) Abundance of selected taxa subjected to fisheries pressure at these three sites. Solid lines connect observed average abundance/biomass per sample per year. Blue dotted lines represent a LOESS smoother to capture the trends in fish abundance. Gray shaded areas show the SE around the mean (dotted line). Gray dashed lines indicate hypothesized directions of response to the anthropause based on anecdotal evidence about human changes in fishing activity. For each site, data presented represent major fisheries at that location: For SBC, data presented are for lobster capture at sites where commercial fishing is permitted (Reed, 2020); for MCR, data reported are in units of fishable biomass compiled for targeted species >15 cm in length (Brooks, 2021); for NTL data reported are from Lake Monona and consist of the top two game species fished in that location, Bluegill and Largemouth Bass, harvested by electrofishing (Magnuson et al., 2019)

We compared three LTER sites with different coupled human–natural systems: (1) the Santa Barbara Coastal LTER is a marine system located on the Pacific coast of southern California, (2) the North Temperate Lakes LTER is a collection of inland, freshwater lakes in North Central Wisconsin, and (3) the Moorea Coral Reef LTER site is located in the Society Island Archipelago of French Polynesia in the central South Pacific Ocean. All three sites experience commercial and/or recreational fishing. However, we expected that the effects of changes in human behavior would differ among them given their different social contexts and this may provide insight into how different locales around the globe may vary in fish population responses to the pandemic (Bennett et al., 2020). At the Santa Barbara Coastal site, there has been a decline in commercial fishing of lobsters within unprotected kelp forest sites due to decreased demand by the restaurant sector (Racino and Meyers, 25 March 2020, “California's fishing industry another victim of coronavirus, including in San Diego,” public communication; https://inewsource.org/2020/03/25/california-commercial-fishing-industry-coronavirus/). By contrast, in the state of Wisconsin where the North Temperate Lakes LTER is located, the Department of Natural Resources reports that the demand for recreational fishing licenses in 2020 increased 30% relative to the previous year (435,000 licenses in 2020 relative to 337,000 licenses in 2019) likely due to an increase in outdoor time, given work‐from‐home orders and the safer environs of open air activities (Langfellow, 29 June 2020, “Wisconsin fishing licenses surge during pandemic,” public communication; https://www.nbc15.com/2020/06/30/wisconsin‐fishing‐licenses‐surge‐during‐pandemic/). Furthermore, in Wisconsin the early pandemic lockdown period overlapped with the start of spring fishing season for target species (e.g., walleye, bass, and pike). Culturally, this is an important time for fishing, as anglers are coming off of a fishing hiatus between ice fishing season and spring spawning. This increase of license sales may not have happened if the initial reactions to the pandemic did not overlap with this culturally exciting fishing time, or occurred in winter, for example. At Moorea Coral Reef, where visitors from abroad have dropped in response to the pandemic, preliminary evidence suggests that locals dependent on a tourism economy facing losses in wages could turn to increased subsistence fishing (S. Holbrook and J. Wencélius, pers. comm.). We anticipate that these changes in human activity could have contrasting impacts on ecological communities through direct and indirect effects (Figure 7).

Changes in fishing intensity during the pandemic could have direct effects on target species including altered demographics and size structures (Russ & Alcala, 1989). More broadly, we would expect to see direct effects on natural populations (i.e., population size and mortality rates from fishing). For example, fish populations could be expected to increase, decrease, or stay about the same depending on how humans typically use a fishery and how that use has changed due to the anthropause (Appendix S2: Figure S3). In contexts in which the anthropause reduced fishing, the pace of population change will be limited by the maximum growth and recruitment rates of fished species. For example, fish biomass was still increasing in protected areas established near the Santa Barbara Coastal LTER site a decade after establishment, emphasizing the time scale of recovery (Caselle et al., 2015). By contrast, population declines due to overfishing can occur very rapidly; global analyses of fisheries collapses indicate that many occur over spans of just 1–2 years (Mullon et al., 2005).

In addition to direct effects, there are a number of indirect effects wherein changes in fishing activity can impact nontarget species resulting in trophic cascades. For instance, long‐term data at Santa Barbara Coastal LTER showed that increased harvest of predators (i.e., lobsters and carnivorous fish) can lead to increase in sea urchin numbers that may lead to changes in macroalgal abundances (Guenther et al., 2012). With decreased commercial fishing due to COVID‐19, we expect that there will be greater top‐down control this year compared to years when fishing pressures are higher. On the coral reefs of Moorea, seaweeds are controlled by herbivorous fishes (Adam et al., 2011) that are targeted by the local small‐scale fishery (Leenhardt et al., 2016; Rassweiler et al., 2020), and research has shown that intensification of fishing (Holbrook et al., 2016) and alteration of fisher behavior (Rassweiler et al., 2021) can reduce resilience of the coral state and promote a switch to seaweed dominated reefs that can represent a difficult‐to‐reverse, alternative basin of attraction (Schmitt et al., 2019, 2021). Relaxation of fishing would strengthen resilience of coral communities to other disturbances such as storms and heat waves (Holbrook et al., 2016).

Our case study of three LTER sites, with different coupled human–natural systems surrounding fisheries, provides a unique opportunity to explore how the spatial scale of human behavior and the connectivity of populations might affect aquatic and marine systems differently. Fished stocks at Santa Barbara Coastal are influenced by local conditions but also by larvae coming from as far away as Mexico (Iacchei et al., 2013), and dynamics at Moorea Coral Reef are likely similarly connected to fisheries beyond the island (Bernardi et al., 2001; Edmunds et al., 2018; Lo‐Yat et al., 2006; Planes et al., 2002). In contrast, there is little natural dispersal of fish between lakes at North Temperate Lakes, but fished species are stocked. These differences in social and ecological contexts can reveal how differences in human behavior interact with underlying differences within the ecologies of these systems. Furthermore, within a single LTER site there can be spatial differences in which a response to the anthropause might be seen in one locale, but not another. For instance, in Moorea different locations within the LTER site have been more or less resilient to coral bleaching events, cyclones, and brief predator outbreaks (Donovan et al., 2020; Holbrook et al., 2018; Kayal et al., 2018). Detecting changes in wildlife in response to the anthropause requires spatial replication for context to determine the generality of findings.

Long‐term research from the LTER sites also illustrates the value of time series for evaluating long‐term trends and anomalous events. The global anthropause lockdown of 2020 was a short‐term pulse disturbance, but one with potentially long‐term benefits to ecosystems. A key question is, how can we confidently attribute any change in wildlife populations to this pulse disturbance as opposed to other drivers? At the three focal sites, there are myriad other drivers causing variability in wildlife populations. For instance, fished species and kelp forest communities in southern California are influenced by strong climate signals at a range of frequencies including the El Niño Southern Oscillation, the North Pacific Gyre Oscillation, and the Pacific Decadal Oscillation (Koslow et al., 2012), whereas fished species at Moorea Coral Reef undergo major shifts in response to natural perturbations such as cyclones (Adam et al., 2014; Holbrook et al., 2008; Rassweiler et al., 2020). In addition to climatic variation, invasive species influence fish communities through direct effects and indirect effects. For instance, at North Temperate Lakes invasive crayfish (*Orconectes* spp.) and rainbow smelt (*Osmerus mordax*) directly compete with fish that use similar resources to them or share prey and indirectly influence other species that respond to changes in community composition caused by them (Willis & Magnuson, 2006). Without long‐term research, it is difficult to distinguish background variability (i.e., underlying temporal turnover) from changes due to these various drivers, including the anthropause.

Replication in space and time is needed in datasets to quantify uncertainty in our ability to attribute change due to the anthropause from background variability or other drivers. The statistical design for such quantification varies depending on the details of each LTER site. For example, Santa Barbara Coastal includes areas open to fishing and also locations in marine protected areas where fishing is restricted. Contrasts between ecological outcomes in the protected and unprotected areas can be used to isolate the cascading effects of fishing. Protected areas in Moorea are not as well enforced, limiting their utility for hypothesis testing, but the diverse fish community includes ecologically similar species, which are valued very differently by fishers (Nassiri et al., 2021). Comparing population trends in targeted and unfished taxa can help reveal the effects of the anthropause in this context. Data on harvested species are currently being collected by these three LTER sites, and time will tell whether or not the hypothesized changes in fisheries occur. Additional research on the social–ecological systems in which these sites are situated would lead to better understanding of the magnitude of changes in fishing that lead to changes in fish populations after accounting for other potential drivers (e.g., Holbrook et al., 2015).

Although the case study of three LTER sites presents time series data based on direct observations of organisms in the field, additional relevant research on wildlife may come from camera trapping or other remotely sensed imagery. Such sources are particularly valuable because it was difficult for many researchers to physically collect data in the field in spring/early summer 2020. Automated digital time‐lapse cameras that capture images across time and space within networks are most likely to be of greatest utility for wildlife studies given the spatial and temporal scale considerations outlined. Some of these camera networks may have been set up with the intention of capturing wildlife images (e.g., Wildlife Camera Network Northwest: https://www.zoo.org/wcnnw) or to test explicit, wildlife‐based ecological hypotheses (e.g., Stears et al., 2020), whereas other networks may capture wildlife images although they are intended for different purposes (e.g., the Phenocam Network, which was designed to monitor vegetation phenology; Brown et al., 2016).

#### Feedback between ecosystem services and societal changes: opportunities revealed during the anthropause

In the previous sections, we discussed how the anthropause may be altering ecosystem structure and function, and how these changes may be uniquely captured through long‐term ecological research. It is well established that ecological processes are inherently scale‐dependent and the various mechanisms rooted beneath ecological patterns operate at different spatial and temporal scales of observation (Levin, 1992). Thus, assessing the effects of the anthropause requires considering the spatial and temporal context of observed changes. The case studies above illustrate the need for long‐term and spatially networked research to distinguish the effects of the anthropause from the influence of other sources of background variation, caused by climate, humans, or other factors, on ecological systems (Smart et al., 2012). In this section, we focus on the feedback of anthropause‐related ecosystem changes to humans and social–ecological systems at scales ranging from the individual to neighborhood to larger regional and national scales.

At smaller scales, increased appreciation of local habitats from backyard gardens to public parks reported during the pandemic (Venter et al., 2020) may provide long‐term benefits to people and ecosystems if behaviors become habitual routine, and low impact (e.g., not overfertilizing) (Kaplan & Kaplan, 1989; Nassauer, 1997). Distributed LTER social–ecological systems research has documented strong links between household income and backyard care that influence biodiversity patterns at larger spatial scales (Wheeler et al., 2017). During the anthropause in the early stage of the pandemic, more people became aware of environmental issues and the needs to access outdoor spaces for maintaining mental health and well‐being, as well as practicing home gardening and urban agriculture for home economic and food security (Khan et al., 2020; Rousseau & Deschacht, 2020). These residential landscape changes could potentially affect productivity, biodiversity, and nutrient flow at much larger scales, which requires further long‐term study. At the same time, residential water use has increased with unintended and inequitable consequences for vulnerable populations who do not have reliable access to clean water (Kakol et al., 2020). The COVID‐19 pandemic has aggravated inequities across society and revealed persistent systemic injustice with considerable long‐term social impacts to communities (Millett et al., 2020), but the long‐term consequences to ecosystems and services remain uncertain. In the United States, COVID‐19 disruptions to conservation research, management, and public engagement in national parks have created opportunities for developing more flexible approaches to monitoring and inclusive methods for virtual public engagements (Miller‐Rushing et al., 2021).

Transformative resilience, described as taking a crisis as a window of opportunity to push for transformative change, may be a long‐term feedback from human society to ecosystems at both local and global scales. *Rebuild Better* or *Building Back Better* is a framework to support equitable and sustainable recovery in the aftermath of any given disaster, including pandemics (Gjerde, 2017; World Health Organization, 2013). Locally, one excellent long‐term outcome of the pandemic would be if cities and towns were to invest in safe outdoor spaces within walking distance of every neighborhood. Regionally, associated with a COVID‐19 economic recovery plan, the European Commission adopted a set of proposals to make the European Union's climate, energy, transport, and taxation policies fit for reducing net greenhouse gas emissions by at least 55% by 2030 (Elliott et al., 2020).

**FIGURE 8:**
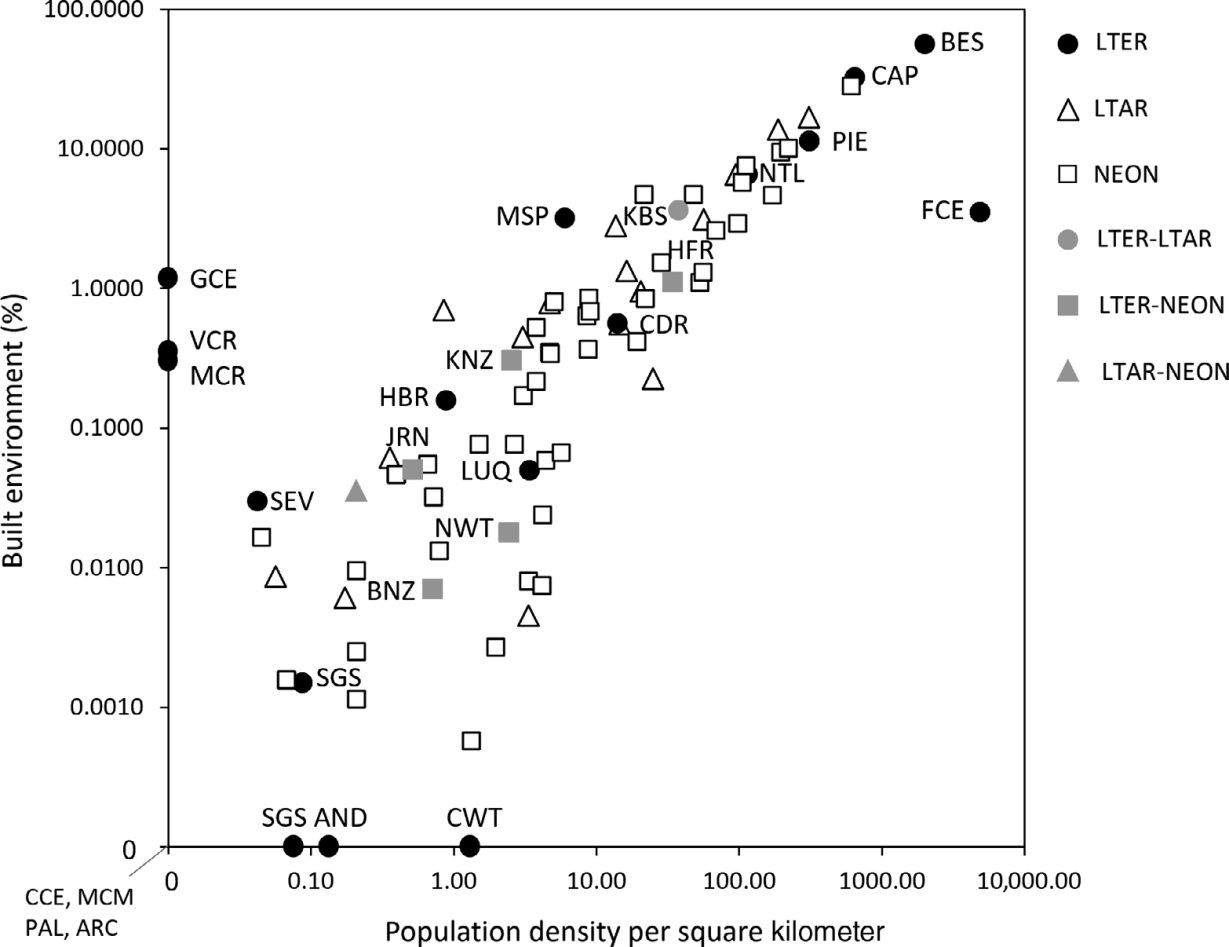
Long Term Ecological Research (LTER), Long Term Agricultural Research (LTAR), and National Ecological Observatory Network (NEON) sites depicted along axes of population density and percent of built environment. Note that data describing population and built environment were not available for marine LTER sites (BLE, NGS, and NGA) and that some sites are members of multiple networks. See Table 1 for the LTER site abbreviation key

Long‐term research plays a critical role in understanding changes at different spatiotemporal scales as exemplified above. Networked research at these larger scales in addition to LTER (such as the National Ecological Observatory Network [NEON] and Long Term Agricultural Research [LTAR] Network) provides the infrastructure and coordination for such spatially and temporally distributed data collection. To illustrate the capacity of LTER, NEON, and LTAR science to collectively inform how social–ecological feedback may influence ecosystem reorganization after the anthropause, we used data from the Global Human Settlement project (Florczyk et al., 2019) to array sites along two human dimensions axes: percent of built area and population density (Figure 8; Appendix S1: Table S1). Generally, sites from all three networks had population densities <310 people per square kilometer and <20% built environment (e.g., impervious surfaces), indicating that the networks are most representative of nonurban areas that may have experienced greater human pressure from recreation during the pandemic. The most densely populated and built‐up sites belonged to the LTER's urban sites (i.e., Central Arizona‐Phoenix and the Baltimore Ecosystem Study). These comparisons show how LTER‐based discoveries of anthropause disturbance impacts might be extended through these partner networks. Data from such coordinated research networks are a key component of ultimately realizing a global monitoring system to support international conservation goals (Pereira et al., 2013). For instance, in situ biodiversity observations from coordinated research networks provide data to ground‐validate maps of essential biodiversity variables generated by remote sensing to inform policy at relevant spatial scales for groups such as the United Nations Framework Convention on Climate Change and the Intergovernmental Panel on Climate Change (Pettorelli et al., 2016). Long‐term data needed to support environmental decision making are increasingly available from many different types of social–ecological systems, creating ample opportunities for refocusing environmental stewardship toward efforts that maximize long‐term resilience to the multiple interacting disturbances of the 21st century.

## CONCLUSIONS

The capacity of people to make environmentally sustainable decisions depends in part on the concept of scale of experience, awareness, willingness, and capacity to act, just as scale matters when investigating other ecological processes. The short human generation time relative to the pace of ecosystem change often compromises the ability to understand and manage complex cross‐scale, long‐term change (Peters et al., 2011; Polasky et al., 2011). A potential societal outcome of the COVID‐19 pandemic is that it helps foster understanding of the personal and local repercussions of a short‐term, global disturbance. Opening the newspaper every day to see people across the globe pictured in masks or having their temperature taken aids in our species' ability to comprehend the effects of individual and collective decision making in the face of a worldwide problem. The anthropause is occurring within the Anthropocene, an epoch when humanity's impact on the world is unprecedented (Crutzen, 2006). A key trait to a resilience mindset is the ability to discover opportunities in the face of adversity (Seery, 2011), which can reveal solutions for moving forward.

Perhaps the pandemic will empower humans to adopt a mindset, which will allow us to become more resilient in the face of global change and help us to recognize the ways in which our collective actions can lead to a more sustainable future for our planet. Ultimately, networked long‐term social–ecological research informs how human systems (e.g., policies, urbanization, built environment, and behaviors) regulate ecosystem resilience, which feeds back to the human system via ecosystem services. Discoveries from networked long‐term science have implications for decision making across scales, disciplines, and governance to achieve resilient and sustainable social–ecological systems from community to global scales.

## CONFLICT OF INTEREST

The authors declare no conflict of interest.

## Supporting information


Appendix S1

Appendix S2
