# Supplementary material for: Long‐term ecological research and the COVID‐19 anthropause: A window to understanding social–ecological disturbance
Source: Ecosphere. 2022 Apr 8;13(4):e4019. doi: 10.1002/ecs2.4019 (PMC9087370; doi:10.1002/ecs2.4019)
Supplement: Supplementary file 1 — Appendix S1 [file ECS2-13-e4019-s002.pdf]

Gaiser, E. E., J. S. Kominoski, D. M. McKnight, C. A. Bahlai, C. Cheng, S. Record, W. M. Wollheim, K. R. Christianson, M. R. Downs, P. A. Hawman, S. J. Holbrook, A. Kumar, D. R. Mishra, N. P. Molotch, R. B. Primack, A. Rassweiler, R. J. Schmitt, and L. Sutter. 2021. Long-term ecological research and the COVID-19 anthropause: A window to understanding social-ecological disturbance. Ecosphere.

Appendix S1

Table S1. Data supporting Figure 8.

**Column**	**Description**
Network	Defines which network(s) the site belongs to (e.g., Long Term Ecological Research Network [LTER], Long Term Agricultural Research Network [LTAR], National Ecological Observatory Network [NEON])
Site	Site name
Site abbreviation	Site name abbreviation for details see respective networks' websites. Two abbreviations are given when sites have different acronyms for the different networks.
Built	Percent of built environment from Global Human Settlement Layer
Population	Population density per square kilometer from the Global Human Settlement Layer

**Network**	**Site**	**Site abbreviation**	**Built**	**Population**
LTAR	Jornada Experimental Range	JER	0.01	0.06
LTAR	Great Basin	GB	0.01	0.17
LTAR	R. J. Cook Agronomy Farm	CAF	0.06	0.36
LTAR	Platte River, High Plains Aquifer	PRHPA	0.70	0.85
LTAR	Archbold-University of Florida	ABS-UF	0.45	3.04
LTAR	Texas Gulf	TG	0.00	3.34
LTAR	Northern Plains	NP	0.79	4.67
LTAR	Upper Mississippi River Basin	UMRB	2.79	13.63
LTAR	Walnut Gulch Experimental Watershed	WGEW	0.56	14.55

LTAR	Southern Plains	SP	1.34	16.27
LTAR	Gulf Atlantic Coastal Plain	GACP	0.96	20.41
LTAR	Lower Mississippi River Basin	LMRB	0.23	24.89
LTAR	Central Mississippi River Basin	CMRB	3.13	56.49
LTAR	Eastern Corn Belt	ECB	6.56	94.99
LTAR	Lower Chesapeake Bay	LCB	13.69	187.09
LTAR	Upper Chesapeake Bay	UCB	16.87	306.38
LTAR-LTER	Kellogg Biological Station	KBS	3.66	37.33
LTAR-NEON	Central Plains Experimental Range	CPER	0.04	0.21
LTER	California Current Ecosystem	CCE	0.00	0.00
LTER	Florida Coastal Everglades	FCE	3.53	4866.00
LTER	Georgia Coastal Ecosystems	GCE	1.11	0.00
LTER	McMurdo Dry Valleys	MCM	0.00	0.00
LTER	Moorea Coral Reef	MCR	0.50	0.00
LTER	Minneapolis-St. Paul	MSP	0.23	419.81
LTER	Palmer Antarctica	PAL	0.00	0.00
LTER	Plum Island Ecosystems	PIE	11.38	310.00
LTER	Santa Barbara Coastal	SBC	1.92	0.00
LTER	Virginia Coast Reserve	VCR	0.54	0.00
LTER	Sevilleta	SEV	0.03	0.04
LTER	Shortgrass Steppe	SGS	0.00	0.09
LTER	Andrews Forest	AND	0.00	0.12
LTER	Hubbard Brook	HBR	0.16	0.88
LTER	Luquillo	LUQ	0.05	3.38
LTER	Coweeta	CWT	0.00	4.33

LTER	Cedar Creek Ecosystem	CDR	0.57	14.03
LTER	North Temperate Lakes	NTL	6.62	114.16
LTER	Central Arizona-Phoenix	CAP	32.44	643.51
LTER	Baltimore Ecosystem Study	BES	56.77	2002.32
LTER-NEON	Arctic/Toolik Field Station	ARC/TOOK	0.00	0.00
LTER-NEON	Jornada Basin/Jornada Experimental Range	JRN/JORN	0.05	0.52
LTER-NEON	Bonanza Creek/Caribou-Poker Creeks Research Watershed	BNZ/BONA	0.01	0.71
LTER-NEON	Niwot Ridge	NIWO	0.02	2.42
LTER-NEON	Konza Prairie Biological Station	KNZ/KONZ	0.31	2.55
LTER-NEON	Harvard Forest & Quabbin Watershed	HFR/HARV	1.11	34.11
NEON	Guanica Forest	GUAN	13.42	0.00
NEON	McRae Creek	MCRA	0.00	0.00
NEON	Oksrukuyik Creek	OKSR	0.00	0.00
NEON	Red Butte Creek	REDB	0.00	0.00
NEON	Smithsonian Environmental Research Center	SERC	7.07	0.00
NEON	Sycamore Creek	SYCA	0.00	0.00
NEON	Blacktail Deer Creek	BLDE	0.00	0.00
NEON	Yellowstone National Park	YELL	0.00	0.00
NEON	Delta Junction	DEJU	0.02	0.05
NEON	Lower Teakettle	TEAK	0.00	0.07
NEON	Teakettle Creek - Watershed 2	TECR	0.00	0.07
NEON	West St Louis Creek	WLOU	0.00	0.07

NEON	Arikaree River	ARIK	0.01	0.21
NEON	Onaqui	ONAQ	0.00	0.21
NEON	Moab	MOAB	0.00	0.21
NEON	Healy	HEAL	0.00	0.33
NEON	Dakota Coteau Field Site	DCFS	0.05	0.40
NEON	Prairie Lake	PRLA	0.05	0.40
NEON	Prairie Pothole	PRPO	0.05	0.40
NEON	Chase Lake National Wildlife Refuge	WOOD	0.05	0.40
NEON	Upper Big Creek	BIGC	0.00	0.41
NEON	Soaproot Saddle	SOAP	0.00	0.41
NEON	McDiffett Creek	MCDI	0.05	0.66
NEON	Caribou Creek	CARI	0.01	0.71
NEON	Mayfield Creek	MAYF	0.03	0.73
NEON	Talladega National Forest	TALL	0.03	0.73
NEON	North Sterling	STER	0.01	0.80
NEON	Little Rock Lake	LIRO	0.00	1.13
NEON	Crampton Lake	CRAM	0.00	1.34
NEON	University of Notre Dame Environmental Research Center	UNDE	0.00	1.34
NEON	Martha Creek	MART	0.08	1.51
NEON	Mountain Lake Biological Station	MLBS	0.00	1.98
NEON	Como Creek	COMO	0.02	2.42
NEON	Kings Creek	KING	0.31	2.55
NEON	Wind River Experimental Forest	WREF	0.08	2.70
NEON	San Joaquin Experimental Range	SJER	0.17	3.09
NEON	Treehaven	TREE	0.01	3.38
NEON	Flint River	FLNT	0.22	3.80

NEON	Lyndon B. Johnson National Grassland	CLBJ	0.52	3.83
NEON	Lenoir Landing	LENO	0.01	4.17
NEON	Lower Tombigbee River	TOMB	0.01	4.17
NEON	Rocky Mountains	RMNP	0.02	4.24
NEON	Abby Road	ABBY	0.06	4.47
NEON	LeConte Creek	LECO	0.35	4.75
NEON	Great Smoky Mountains National Park	GRSM	0.35	4.76
NEON	Bartlett Experimental Forest	BART	0.34	4.79
NEON	Santa Rita Experimental Range	SRER	0.80	5.11
NEON	Steigerwaldt-Chequamegon	STEI	0.07	5.71
NEON	Blue River	BLUE	0.63	8.65
NEON	Marvin Klemme Range Research Station	OAES	0.36	8.81
NEON	The Jones Center At Ichauway	JERC	0.85	8.89
NEON	Pringle Creek	PRIN	0.68	9.07
NEON	Lower Hop Brook	HOPB	0.41	19.31
NEON	Oak Ridge	ORNL	4.65	21.67
NEON	Walker Branch	WALK	4.65	21.67
NEON	Blandy Experimental Farm	BLAN	0.84	22.27
NEON	Lewis Run	LEWI	0.84	22.27
NEON	Lake Barco	BARC	1.52	28.62
NEON	Lake Suggs	SUGG	1.52	28.62
NEON	Black Warrior River	BLWA	4.64	48.25
NEON	Dead Lake	DELA	4.64	48.25
NEON	Rio Guilarte	GUIL	1.10	53.76
NEON	Rio Cupeyes	CUPE	1.29	56.16
NEON	Ordway-Swisher Biological Station	OSBS	2.59	69.02

NEON	Posey Creek	POSE	2.91	98.21
NEON	Smithsonian Conservation Biology Institute	SCBI	2.91	98.21
NEON	University of Kansas Field Station	UKFS	5.73	106.00
NEON	Northern Great Plains Research Laboratory	NOGP	7.51	113.16
NEON	Utqiaġvik	BARR	4.63	172.36
NEON	Disney Wilderness Preserve	DSNY	9.38	198.01
NEON	Lajas Experimental Station	LAJA	10.05	222.55
NEON	Konza Prairie Agroecosystem	KONA	27.79	618.52
