# Supplementary material for: Long‐term ecological research and the COVID‐19 anthropause: A window to understanding social–ecological disturbance
Source: Ecosphere. 2022 Apr 8;13(4):e4019. doi: 10.1002/ecs2.4019 (PMC9087370; doi:10.1002/ecs2.4019)

Gaiser, E. E., J. S. Kominoski, D. M. McKnight, C. A. Bahlai, C. Cheng, S. Record, W. M. Wollheim, K. R. Christianson, M. R. Downs, P. A. Hawman, S. J. Holbrook, A. Kumar, D. R. Mishra, N. P. Molotch, R. B. Primack, A. Rassweiler, R. J. Schmitt, and L. Sutter. 2021. Long-term ecological research and the COVID-19 anthropause: A window to understanding social-ecological disturbance. Ecosphere.

Appendix S2

Figure S1. Application of the disturbance framework to the effects of the anthropause on the lakes of the Niwot Ridge LTER (Case Study 1).

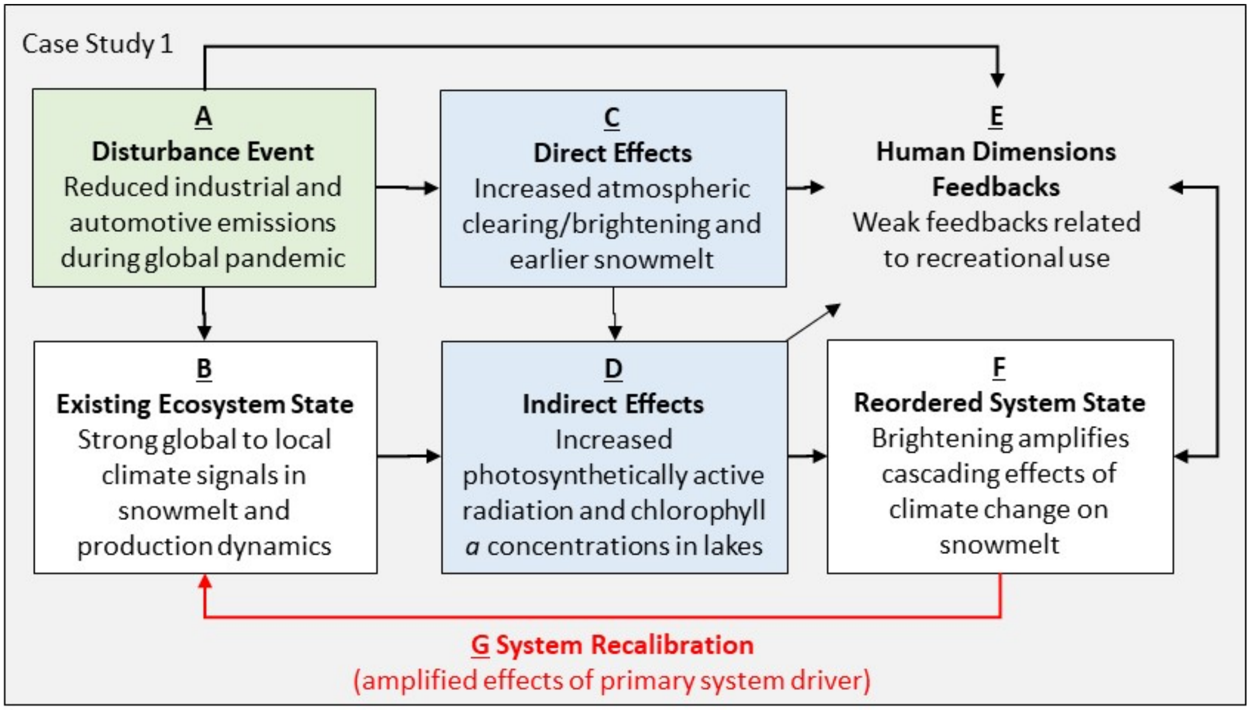

Figure S2. Application of the disturbance framework to the effects of the anthropause on Georgia Coastal Ecosystems LTER (Case Study 2).

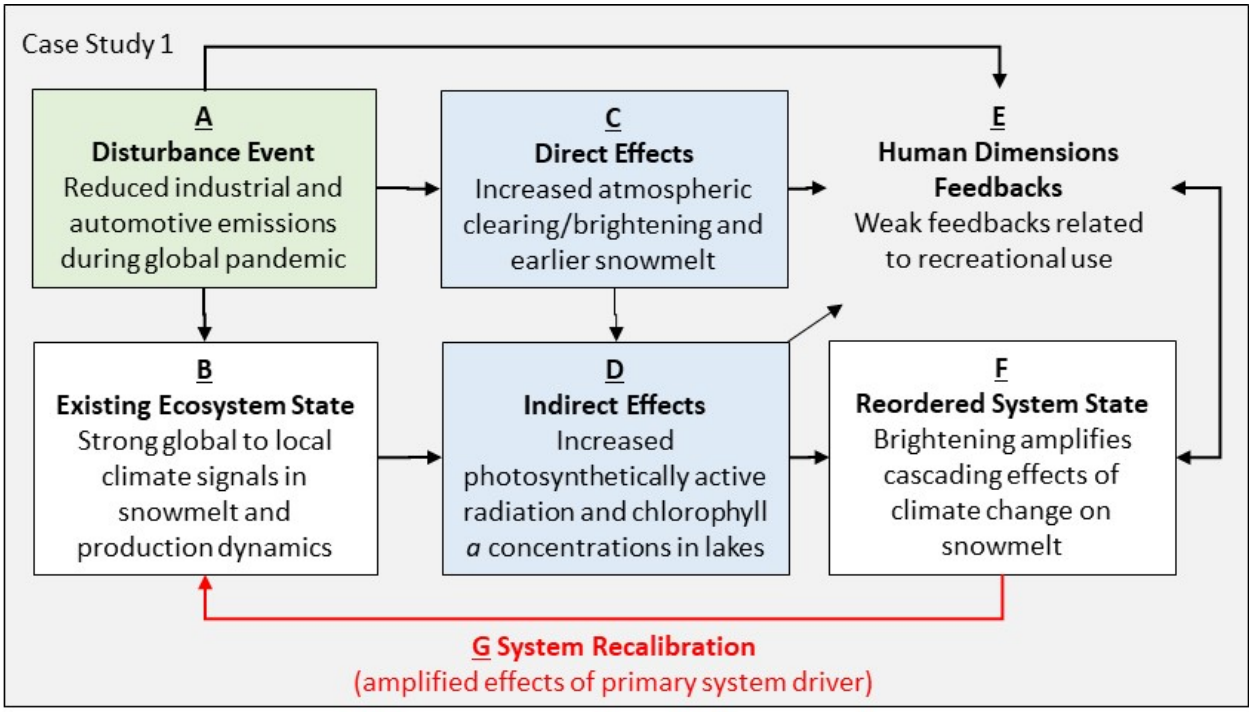

Figure S3. Application of the disturbance framework to the effects of the anthropause on recreational and commercial fishing and trophic dynamics (Case Study 3).

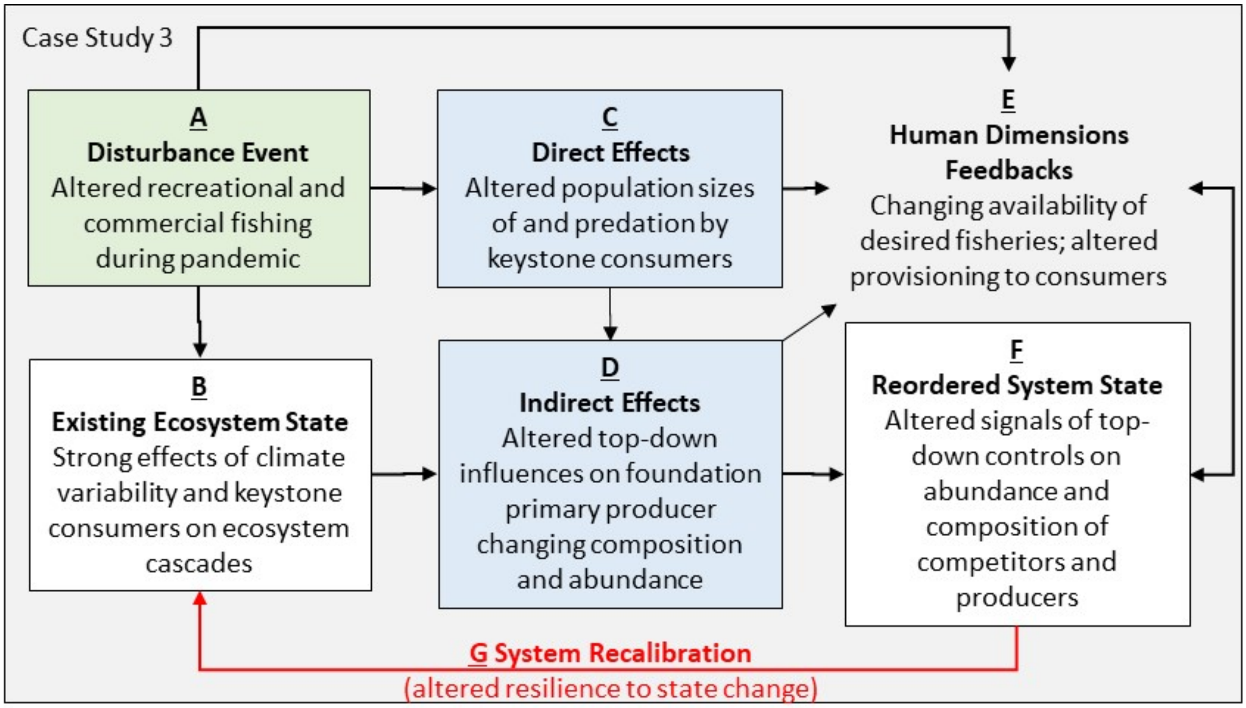

Supplement: Supplementary file 2 — Appendix S2 [file ECS2-13-e4019-s001.pdf]
